# Analysis of Aminoglycoside Antibiotics: A Challenge in Food Control

**DOI:** 10.3390/molecules28124595

**Published:** 2023-06-07

**Authors:** Ewelina Nowacka-Kozak, Anna Gajda, Małgorzata Gbylik-Sikorska

**Affiliations:** Department of Pharmacology and Toxicology, National Veterinary Research Institute, Partyzantów 57, 24-100 Puławy, Poland; anna.gajda@piwet.pulawy.pl (A.G.); malgorzata.gbylik@piwet.pulawy.pl (M.G.-S.)

**Keywords:** aminoglycoside antibiotics, UHPLC-MS/MS, analysis, food, residues

## Abstract

Aminoglycosides are a widely used group of antibiotics in veterinary medicine. However, misuse and abuse of these drugs can lead to residues in the edible tissues of animals. Due to the toxicity of aminoglycosides and the exposure of consumers to the emergence of drug resistance, new methods are being sought to determine aminoglycosides in food. The method presented in this manuscript describes the determination of twelve aminoglycosides (streptomycin, dihydrostreptomycin, spectinomycin, neomycin, gentamicin, hygromycin, paromomycin, kanamycin, tobramycin, amikacin, apramycin, and sisomycin) in thirteen matrices (muscle, kidney, liver, fat, sausages, shrimps, fish honey, milk, eggs, whey powder, sour cream, and curd). Aminoglycosides were isolated from samples with extraction buffer (10 mM NH_4_OOCH_3_, 0.4 mM Na_2_EDTA, 1% NaCl, 2% TCA). For the clean-up purpose, HLB cartridges were used. Analysis was performed using ultra-high-performance liquid chromatography coupled with tandem mass spectrometry (UHPLC-MS/MS) with a Poroshell analytical column and a mobile phase of acetonitrile and heptafluorobutyric acid. The method was validated according to Commission Regulation (EU) 2021/808 requirements. Good performance characteristics were obtained for recovery, linearity, precision, specificity, and decision limits (CCα). This simple and high-sensitivity method can determine multi-aminoglycosides in various food samples for confirmatory analysis.

## 1. Introduction

Aminoglycosides are the oldest classes of antimicrobials. These are weak bases but highly polar and poorly soluble in lipids due to sugar residues in the molecules. These compounds characteristically contain several amino sugars connected to glycosidic bonds to an aminocyclitol component [1]. Aminoglycosides can be divided into four classes based on identity on aminocyclitol: (I) no deoxystreptamine (streptomycin); (II) a mono-substituted deoxystreptamine ring (apramycin); (III) a 4,5-di-substituted deoxystreptamine ring (neomycin, paromomycin, ribostamycin); and (IV) a 4,6-di-substituted deoxystreptamine ring (gentamicin, amikacin, tobramycin, kanamycin). Aminoglycosides can consist of several different chemical compounds, for example, neomycin (two stereoisomers: B, C), gentamycin (C1, C1A, C2A, and C2B), paromomycin (two stereoisomers: I, II), and kanamycin (3 isomers: A, B, and C) [2].

Aminoglycoside antibiotics are among the most common antibiotics in animal husbandry to treat severe bacterial infections. They are applicable against infections caused by Gram-negative and also Gram-positive organisms. Improper use of drugs, failure to observe the withdrawal period, or slaughtering animals during treatment can result in drug residues in the tissues. Due to extended withdrawal times and high residue levels, using these drugs as veterinary medicines carries the risk of developing resistance among bacteria, which may reduce the effectiveness of these drugs as human medicines [3]. Nearly all of the aminoglycosides are widely used in human and veterinary medicine. The residues of aminoglycoside antibiotics in food of animal origin may represent a risk to consumer health because of possible allergenicity and toxicity. Despite their high antimicrobial efficiency, aminoglycosides can accumulate in tissues and damage the nervous and digestive systems. These drugs may cause toxic effects such as ototoxicity; nephrotoxicity; fetal damage (passing through the placenta); and rare, neuromuscular blockade and hypersensitivity reactions [1,4].

As part of the EU’s official monitoring programs, mandatory monitoring of aminoglycosides in food of animal origin is carried out to ensure food safety and consumer health. The European Commission has established strict maximum residue levels (MRLs) for some aminoglycosides in various matrices (muscle, fat, liver, kidney, milk, eggs), which are included in Commission Regulation (EU) No. 37/2010 of 22 December 2009, on pharmacologically active substances and their classification regarding maximum residue limits in foodstuffs of animal origin [5]. The values of aminoglycoside MRLs range from 50 µg/kg for gentamicin in bovine muscle and fat to 20,000 µg/kg for apramycin in bovine kidney (Table 1). However, no MRLs have been specified for honey in the EU, except streptomycin, for which the Community Reference Laboratory in France has set a recommended concentration of 40 µg/kg [6,7]. The regulation does not include amikacin, hygromycin B, sisomycin, and tobramycin. In addition, MRLs in milk have not been established for apramycin and paromomycin, while in eggs, the MRL values are only for neomycin and paromomycin. The lack of assigned MRL values for the remaining compounds requires the development of a sensitive method for determining aminoglycosides.

Every year, the European Food Safety Authority (EFSA) publishes a report presenting the results of monitoring residues of veterinary drugs and other substances in live animals and animal products. The last 10 EFSA reports (2012–2021) [8,9,10,11,12,13,14,15,16,17] show that the percentage of aminoglycosides in non-compliant results from the entire group B1 (antimicrobial substances, including sulfonamides, quinolones) is more than 26%. There was a total of 489 results with aminoglycosides within ten years. The most frequently detected aminoglycoside was dihydrostreptomycin (291 non-compliant results), gentamicin (91 non-compliant results), and neomycin (58 non-compliant results). The others were spectinomycin (30 non-compliant results), streptomycin (12 non-compliant results), paromomycin (4 non-compliant results), and kanamycin (3 non-compliant results). Considering the animal species, the highest numbers of non-compliant results were found in the muscles of cattle (300) and pigs (119). There were 33 non-compliant results in sheep and goats; 16 in poultry; and 1 result each in aquaculture, horses, and rabbits. In honey, there were 10 non-compliant results: streptomycin and dihydrostreptomycin. In milk, there were eight non-compliant results containing dihydrostreptomycin, gentamicin, paromomycin, or kanamycin. It is important to note that the number of samples with non-compliant results has decreased significantly. While in 2012 and 2013, there were 83 and 76 non-compliant results, in 2020 and 2021, there were only 17 and 18, respectively.

The Rapid Alert System for Food and Feed (RASFF) has recorded one notification in the past ten years. It was dihydrostreptomycin in honey (267 µg/kg) in 2021. Some factors, such as high polarity, lack of chromophores or fluorophores, polycationic character, and low volatility, make a challenging task at the sample preparation and final determination stages [18]. Additionally, most of them consist of compounds with closely similar structures. It would affect the difficulty of including this group of compounds in multi-component methods with other antibiotics. Therefore, in the available literature, only a few publications in which aminoglycosides are included in multi-class methods [19,20]. Glinka et al. [21] reviewed the data about sample preparation and clean-up procedures for determining aminoglycosides in various matrices. Moreover, Li et al. [22] have collected information on aminoglycoside detection techniques. However, in most of the literature, aminoglycosides are determined only in two or three matrices such as muscle, eggs, milk [23]; muscles, liver, kidney [24,25]; honey and royal jelly [7]; honey and milk [26]; muscle, honey, milk [27]; or wastewater samples [28]. Various sample purification techniques are described in the literature, such as ion-exchange SPE sorbents [7,29], hydrophilic–lipophilic balance SPE sorbents [25,26,30], and non-polar SPE sorbents [31]. LC-MS/MS was described as the most common detection method [24,26,32,33,34,35]. The methods presented were mainly concerned with a few aminoglycosides. So far, the largest number of analytes in the method (21) has been presented by Kim et al. [23] only in muscle, egg, and milk. Moreover, Yang et al. [36] described some aminoglycosides in honey, milk, meat, and liver. To date, no methods allow for simultaneous determination of aminoglycosides in the muscle, kidney, liver, fat, sausages, shrimps, fish honey, milk, eggs, whey powder, sour cream, and curd.

This study aimed to develop one all-purpose method for food matrices. It is the first time an analytical method has been described for determining twelve aminoglycoside antibiotics in a wide range of matrices (honey, eggs, tissues, meat, and milk products) not presented in other publications. In addition, a significant challenge was the determination of one method of compounds without MRLs (low decision limit (CCα) required) as well as compounds with high MRL values (wide range of method linearity required).

## 2. Results and Discussion

### 2.1. Optimization of LC-MS/MS Conditions

Aminoglycosides are polar compounds due to several amino groups contributing to their weak basic nature [37]. Confirming the presence of aminoglycosides at trace residue concentrations using ultra-high-performance liquid chromatography coupled with the tandem mass spectrometry (UHPLC-MS/MS) method has been a challenge for a long time. A notable problem was the separation of aminoglycosides since some have similar structural forms and mass, such as DHSTR, STR, PAR, and NEO. Detection was performed in multiple reaction monitoring (MRM) modes by directly infusing the standard analyte solution into the mass spectrometer. Two transitions were monitored for each analyte, except ribostamycin as an internal standard with one transition. Three transitions were monitored for aminoglycosides with similar retention times or the same ions for better identification and chromatographic separation. The additional transitions were performed for AMI, DHSTR, KAN, NEO, PAR, and STR for quantification to serve the most abundant precursor ion transitions. In contrast, other transitions have been used for identification. The following mass spectrometry parameters, in positive ionization mode, were designated: collision energy (CE), declustering potential (DP), dwell time, and cell exit potential (CXP) of each compound (Table 2).

To achieve optimal separation of aminoglycosides and UHPLC-MS/MS quantification as well as confirmation, reversed-phase C18 chromatographic columns were tested—Poroshell 120 EC-C18 (2.1 × 50 mm^2^; 2.7 µm), Agilent ZORBAX SB-C18 (50 mm × 2.1 mm, 1.8 µm), and Poroshell 120 EC-C18 (2.1 × 150 mm^2^; 2.7 µm). The separation of aminoglycosides on the first column gave unsatisfactory results; non-symmetrical peaks were observed, particularly for GEN, SPC, and PAR. Moreover, low-intensity peaks on the ZORBAX SB-C18 column were obtained, especially for DHSTR, KAN, GEN, APR, and TOB.

The most efficient way to obtain suitable retention of these polar compounds is to use ion-pairing (IP) agents. Błądek et al. [38] compared various IP reagents, namely, heptafluorobutyric acid (HFBA), trifluoroacetic acid (TFA), and pentafluoropropionic acid (PFPA). During the optimization of chromatographic separation, different IP agents as a mobile phase (TFA, HFBA) with various combinations of gradient programs were tested. In this study, in our method, HFBA in low concentration gave the highest ionization, the best shapes of peaks, good recoveries, and a short elution time. In many multi-component works, HFBA is used as an ion-pairing agent [10,39,40]. The following parameters were tested during chromatographic optimization: the chromatographic column’s temperature and the mobile phase’s flow. Next, temperature was checked: 30 °C, 35 °C, and 40 °C. Different values of flow rate were optimized: 0.25, 0.30, 0.35, and 0.4 mL/min.

**Figure 1:**
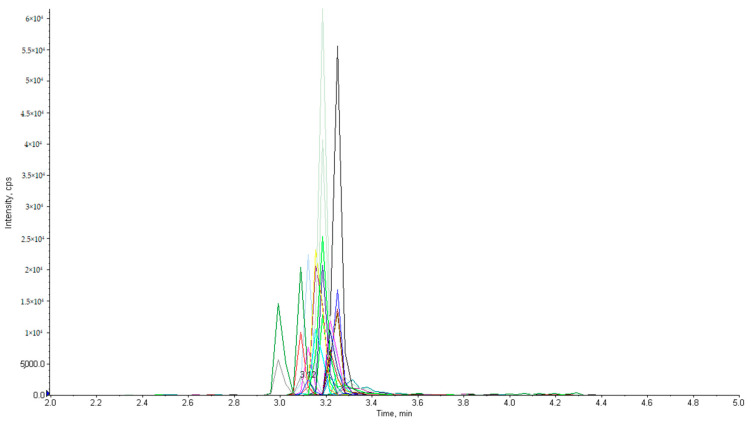
Chromatogram of a standard sample of 12 aminoglycosides at a 0.2 µg/mL concentration.

Finally, the most satisfactory results to analyze AMI, APR, DHSTR, GEN, HYG, KAN, NEO, PAR, SIS, SPC, STR, and TOB were achieved using a longer Poroshell 120 EC-C18 (2.1 × 150 mm; 2.7 µm) column at a temperature of 35 °C with acetonitrile and 0.025% HFBA mobile phase. The flow rate was set as 0.3 mL/min. The chromatographic separation of 12 standards of aminoglycosides is presented in Figure 1.

### 2.2. Optimization of Sample Preparation

**Figure 2:**
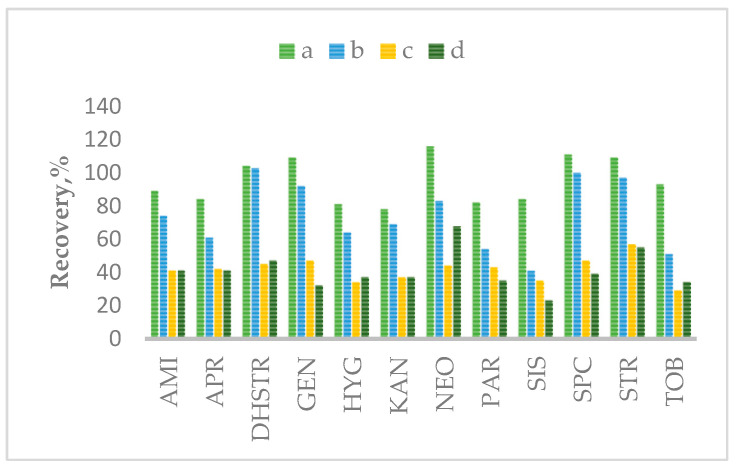
The effect of different extraction mixtures: a—10 mM ammonium acetate/0.4 mM EDTA/1% NaCl/2% TCA with 0.2 µL 0.3 M HFBA; b—10 mM ammonium acetate/0.4 mM EDTA/1% NaCl/2% TCA with 0.5 µL 0.3 M HFBA; c—150 mM EDTA + 15% TCA; d—150 mM EDTA + 5% TCA.

Based on Cherlet et al. [32,41], some extraction mixtures, in various volumes and concentrations, were tested: 10 mM ammonium acetate/0.4 mM EDTA/1% NaCl/2% TCA with 0.2 µL/0.5 µL 0.3 M HFBA; 150 mM EDTA + 5%/15% TCA. The mainly used chemical reagents for sample preparation are TCA + EDTA [32,42,43], NH_4_OOCH_3_ + Na_2_EDTA + NaCl + TCA, and K_2_HPO_4_ in various concentrations [30]. The best results for all aminoglycoside isolations from the matrix were obtained in the presented method after using a solution consisting of 10 mM ammonium acetate/0.4 mM EDTA/1% NaCl/2% TCA. Different TCA concentrations in the extraction solution were also tested (1%, 2%, 5%, 15%). Figure 2 shows the recovery (%) of all analyzed aminoglycosides after using different extraction mixtures.

**Figure 3:**
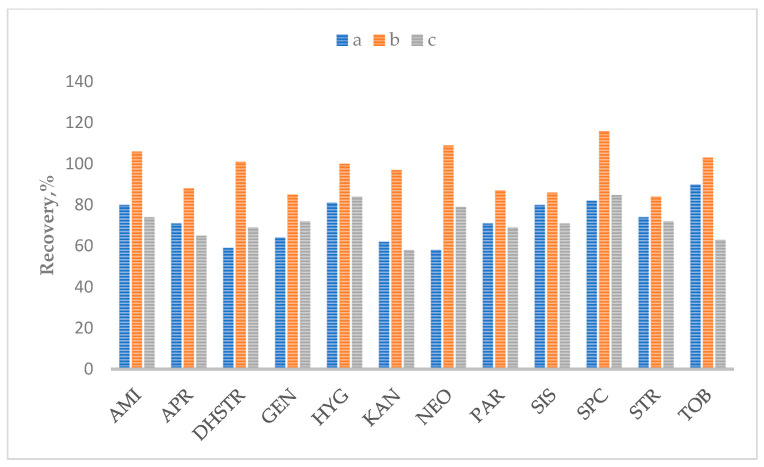
The effect of different pH values: a—pH = 5.5; b—pH = 6.5; c—pH = 8.0, in the liver.

**Figure 4:**
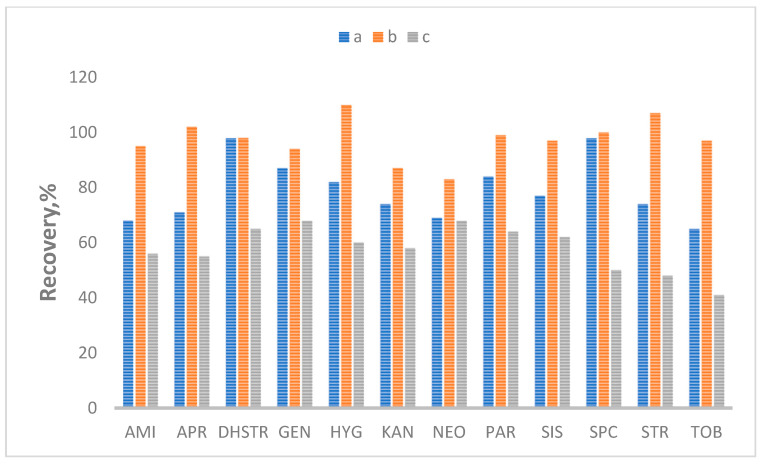
The effect of different pH values: a—pH = 5.5; b—pH = 6.5; c—pH = 8.0, in honey.

The very high sensitivity of aminoglycosides to change the pH value in the extraction solution makes selecting sample preparation conditions a significant challenge for the analyst. Thus, different pH values were tested (5.5, 6.5, and 8.0). Figure 3 and Figure 4 show the effect of pH values for liver and honey samples as the most sensitive matrices. The results for other matrices are presented in the Supplementary Materials (Figure S1). Tests show that at pH = 8.0, the peak area decreased by 50–80% in most cases. These results confirm that aminoglycosides are sensitive to pH changes and that pH = 6.5 is optimal for determining most aminoglycosides.

The next step was to select the appropriate cleaning up. Generally, extraction methods published for aminoglycoside analysis mostly used SPE extraction with Oasis HLB or Strata X cartridges. Oasis HLB is used in many laboratories because of its good retention properties and highly reproducible recovery of a wide range of compounds, both polar and non-polar (due to the combination of their hydrophobic–hydrophilic retention mechanism). In this study, Strata X (100 mg, 6 mL), Strata X-CW (100 mg, 6 mL)—cation weak, Strata X-AW (100 mg, 6 mL)—anion weak, and Oasis HLB (60 mg, 3 mL) cartridges were verified (Table 3). Strata X-CW gave the worst results for most of the matrices. The results were entirely satisfactory only in muscle, liver, and eggs. Strata X, in turn, gave the best results in honey and good results in kidney, fat, sausages, milk, and whey powder. The results between Strata X-AW and Oasis HLB cartridges were comparable for some matrices such as liver, honey, and milk. The Strata X-AW gave significantly better results for eggs, fish, and shrimps. However, the number of compounds and matrices makes it necessary to find a single optimal solution, so after analyzing all the columns, it was decided to use Oasis HLB cartridges with the best results for all tested compounds in all matrices. For better recovery of analytes, a double elution was used with the mixture of formic acid/isopropanol/water (10:5:85).

### 2.3. Method Validation

**Figure 5:**
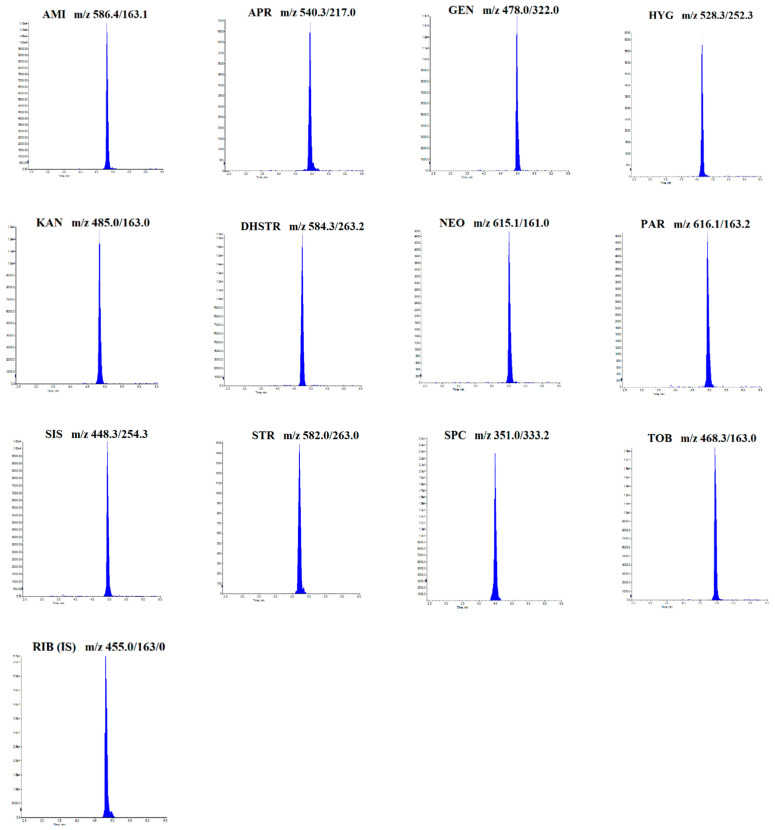
Chromatograms of a shrimp sample fortified with 13 aminoglycosides at 100 µg/kg (2 VL).

The developed method was validated according to the Commission Implementing Regulation (EU) 2021/808 [44]. A good linearity was obtained, and the correlation coefficients (r2) were higher than 0.98 for all analytes in each matrix. The selectivity of the test showed no interference peaks in the samples analyzed. Moreover, repeatability (CVs within 3.5 to 14.2, depending on compound and matrix) and within-laboratory reproducibility (CVs within 4.3 to 13.8, depending on compound and matrix) were satisfactory. The recovery values ranged from 82% to 118% for trueness −79–117%. For ruggedness, the factors and changes examined did not affect the results, indicating that the method developed is robust to minor changes that may occur in the study. The limits of quantifications (LOQ) were from 10 µg/kg to 250 µg/kg. The coefficients of variations are under 20% for the matrix factor (MF) in every analyte and matrix. The summary of validation is shown in Table 4. The example chromatograms (MRM) of a shrimp sample, as a matrix without MRL, fortified at a validation level (VL) of 100 µg/kg with all twelve analytes are presented in Figure 5.

### 2.4. Aminoglycosides in Real Samples

Nearly 750 muscle, tissue, and honey samples were tested for confirmation by LC-MS/MS methods over the past ten years in our laboratory. Of these, 129 samples (about 17%) contained aminoglycosides, of which up to 61% were non-compliant. The most frequently detected aminoglycoside antibiotics were DHSTR (49 non-compliant results, 19 compliant results) in the concentration range 60–229,000 µg/kg and NEO (17 non-compliant results, 22 compliant results) in the concentration range 256–87,000 µg/kg. Aminoglycosides were mainly detected in cattle (96 results) and swine (17 results). Although it may seem that the percentage of samples with aminoglycosides is small compared to other groups (tetracyclines), the toxicity of these compounds and the difficulty of quantifying them seems to be quite a challenge for analysts.

The method presented in this paper has been implemented for the official analyses of aminoglycosides in eggs as a part of the National Residue Control Plan for the surveillance of veterinary drug residues in food of animal origin as well as in commercial research. So far, no aminoglycosides have been detected in any of the egg samples tested. Additionally, the method described in this study determining 12 aminoglycosides was verified by analyzing the confirmation samples sent in the last year. More than 20 muscle and kidney samples from cattle and pigs were analyzed. Seven kidney samples contained aminoglycosides (NEO, DHSTR) at a concentration of 794–21,950 µg/kg, of which three were non-compliant results above CCα. The demonstrated method will be applied in the future to official control of aminoglycosides in other matrices, as the method developed so far does not cover the need for analyses of new compounds (AMI, APR, HYG, SIS, TOB) and matrices (processed food).

## 3. Materials and Methods

### 3.1. Chemical and Reagents

HPLC-grade acetonitrile, methanol, and isopropanol were purchased from J.T. Baker (Deventer, The Netherlands). Ethylenediaminetetraacetic acid (EDTA), sodium chloride, and sodium hydroxide were from POCH (Gliwice, Poland), and potassium hydrogen phosphate (K_2_HPO_4_) was from Chempur (Piekary Śląskie, Poland). Ammonium acetate, heptafluorobutyric acid (HFBA), and trichloroacetic acid (TCA) were obtained from Sigma–Aldrich, (St. Louis, MO, USA). Formic acid was from Fluka (Charlotte, NC, USA). Syringe 0.22 µm hydrophilic polyvinylidene fluoride (PVDF) membrane filters were purchased from Restek (Bellefonte, PA, USA). Ultra-pure water was generated by a Millipore Milli-Q System (Millipore, Molsheim, France).

The analytical reference standards of DHSTR, GEN, KAN, NEO, PAR, STR, and SPC were purchased from Dr Ehrenstorfer (Augsburg, Germany), and AMI, APR, HYG, RIB, SIS, TOB from Sigma–Aldrich (St. Louis, MO, USA). Strata X (100 mg, 6 mL), Strata X-CW (100 mg, 6 mL), and Strata X-AW (100 mg, 6 mL) cartridges were from Phenomenex (Torrance, CA, USA). Oasis HLB (60 mg, 3 mL) cartridges were from Waters (Milford, MA, USA). All matrices were obtained from local supermarkets or were originating from the Polish official residue control program. Samples were analyzed to ensure the absence of aminoglycoside residues and kept frozen at −18 °C until use.

### 3.2. Preparation of the Standard Stock Solution and Working Solutions

Each analyte’s individual standard stock solutions (1000 µg/mL) were dissolved in a mixture of water/acetonitrile/acetic acid (78:20:2, *v*/*v*/*v*). All standard stock solutions were stored at −18 °C for no longer than 3 months. Working solutions and an internal standard solution (IS) were prepared in ultra-pure water and stored at 4 °C for 1 month.

### 3.3. Sample Preparation

A 1.00 ± 0.01 g of homogenized samples (muscle, kidney, liver, fat, sausages, shrimps, fish, honey, milk, eggs, whey powder, sour cream, and curd) were weighed into a 50 mL polypropylene centrifuge tube. The 20 µL of the internal standard (20 µg/mL) was added. Then, 10 mL of extraction buffer (10 mM NH_4_OOCH_3_/0.4 mM Na_2_EDTA/1% NaCl/2% TCA) was added. Samples were vortex mixed, rotary shaken (10 min), and centrifuged (4500 rpm, 10 min, 4 °C). The pH of the supernatants was adjusted to 6.5 with 1 M NaOH solutions. Next, samples were put on Oasis HLB (60 mg, 3 mL) cartridges preconditioned with 5 mL of methanol and 5 mL of water. Aminoglycosides were eluted with a mixture: of formic acid/isopropanol/water (10:5:85), twice each at 500 µL. Finally, 200 µL of 0.3 M HFBA was added to the final extract, and after filtration using 0.22 µm PVDF filters, the extracts were moved to chromatographic glass vials and analyzed by UHPLC-MS/MS.

### 3.4. UHPLC-MS/MS Analysis

Aminoglycosides were analyzed using ultra-high-performance liquid chromatography Shimadzu Nexera X2 (Shimadzu, Kyoto, Japan) connected to the QTRAP 4500 triple quadrupole mass spectrometer (Sciex, Framingham, MA, USA). The Analyst 1.6.2 software (Sciex, Framingham, MA, USA) processed the data. The mass spectrometry detection was operated in the positive ESI mode with multiple reaction monitoring. The temperature of desolvation was set at 450 °C, ion spray voltage: 4500 V, nebulizer gas (N_2_): 60 psi, curtain gas (N_2_): 20 psi, collision gas (N_2_): medium, auxiliary gas (N_2_): 65 psi. All MS/MS parameters are presented in Table 2.

Chromatographic separation was achieved on a Poroshell 120 EC-C18 (2.1 × 150 mm; 2.7-µm) analytical column (Agilent Technologies, Santa Clara, CA, USA) connected to an octadecyl guard column (4 mm × 2 mm) (Phenomenex, Torrance, CA, USA), operated at 35 °C at a flow rate of 0.3 mL/min. Acetonitrile (B) and 0.025% HFBA (A) was used as a mobile phase in gradient mode. The elution program started from 90% of solvent A (0.01–4.00 min), then decreased to 20% (4.01–5.30 min), and finally came back to 90% (5.31–7.00 min). The injection volume was 5 µL, and the total run time was 7 min.

### 3.5. Method Validation

The presented method was validated according to the Commission Implementing Regulation (EU) 2021/808 of 22 March 2021, repealing Decisions 2002/657/EC and 98/179/EC. The validation process consisted of determining the following parameters: linearity, selectivity, precision, recovery, and decision limit (CCα). The limit of quantification (LOQ) and matrix effect were also assigned. Linearity was determined using a matrix-matched calibration curve prepared by fortifying antibiotic-free matrices at ten concentration levels, depending on the analyte and matrices. Twenty blank samples for different matrices were analyzed for potential disruption with endogenous substances to determine the selectivity. Precision (repeatability and within-laboratory reproducibility) was determined after fortifying six samples on 1, 2, and 3× VL for matrices without MRL, and in the case of designated values of MRL—0.1–0.5, 1, 1.5 MRL, in six replicates at each level. The repeatability was carried out on the same day, instrument, and operator. The coefficient of variations (CV) was calculated. Within-laboratory reproducibility was determined on two days with the same instrument and operators. For trueness, samples were fortified as for precision (1, 2, and 3× VL for matrices without MRL, and in the case of designated values of MRL—0.1–0.5, 1, 1.5 MRL) in six replicates. Trueness (%) was calculated as (mean recovery-corrected concentration detected) × 100/fortification level. The overall CVs were calculated. To test the method’s ruggedness, we tested the sample centrifugation temperature, chromatography column temperature, volume of extraction mixture, and concentration of HFBA added to the final extract. The results were analyzed using the Youden test. Decision limits were determined by analyzing 20 blank samples fortified above the MRL (for authorized substances) or VL (for unauthorized substances). The LOQ was established as the lowest point of the matrix calibration curve. The relative matrix effect was calculated for 20 different blank samples at VL. The matrix effect was assessed by calculating the matrix factor (MF) as the ratio of the analytes peak area of the extract fortified after extraction relative to the peak area obtained from the standard solution. The relative matrix effect was calculated as MF (standard) = peak area of MMS standard/peak area of solution standard, where MMS is matrix-matched standard. The calculated CV should not be greater than 20%.

## 4. Conclusions

Despite the current trends in the use of newer and less toxic antibiotics, aminoglycosides are still very popular due to their relatively low cost and wide range of action. Awareness is growing about the risks to consumer health of consuming food contaminated with antibiotic residues. Therefore, it is important to control their use with validated and sensitive methods developed for detecting residues in food of animal origin.

As indicated in the literature, this is the very first study on the simultaneous determination of aminoglycosides in as many as thirteen different matrices (muscle, kidney, liver, fat, sausages, shrimps, fish, honey, milk, eggs, whey powder, sour cream, curd). Even though the matrices were different, it was possible to match one technique of aminoglycosides extraction in all tested food materials. Satisfactory validation results confirm that the developed method can be used to analyze aminoglycosides as a part of the National Residue Control Plan for surveillance of veterinary drug residues in food of animal origin.

## Figures and Tables

**Table 1:** Maximum residue limits (MRL) for aminoglycosides in food of animal origin according to the Commission Regulation EU No. 37/2010.

Aminoglycoside	Food Origin (Animal Species)	MRL (µg/kg)
Apramycin	Bovine	1000 (muscle, fat) 10,000 (liver) 20,000 (kidney)
Dihydrostreptomycin	All ruminants, porcine, rabbit	500 (muscle, fat, liver) 1000 (kidney) 200 (ruminants’ milk)
Gentamicin	All mammalian food producing species and fin fish	50 (muscle, fat) 200 (liver) 750 (kidney) 100 (milk)
Kanamycin	All food-producing species except fin fish	100 (muscle, fat) 600 (liver) 2500 (kidney) 150 (milk)
Neomycin	All food-producing species	500 (muscle, fat, except fish) 5500 (liver, except fish) 9000 (kidney, except fish) 1500 (milk) 500 (eggs)
Paromomycin	All food-producing species	500 (muscle) 1500 (liver, kidney; except fish) 200 (eggs)
Spectinomycin	Ovine	300 (muscle) 500 (fat) 2000 (liver) 5000 (kidney) 200 (milk)
	All other food-producing species	300 (muscle) 500 (fat, except fish) 1000 (liver, except fish) 5000 (kidney, except fish) 200 (milk)
Streptomycin	All ruminants, porcine, rabbit	500 (muscle, fat, liver) 1000 (kidney) 200 (ruminants milk)

**Table 2:** Mass spectrometry parameters for the quantitative analysis of aminoglycoside antibiotics: collision energy (CE), declustering potential (DP), dwell time, and cell exit potential (CXP).

Analyte	Parent Ion (*m*/*z*)	Daughter Ion(s) (*m*/*z*)	Retention Time (min)	DP (V)	CE (eV)	CXP (eV)
Amikacin (AMI)	586.4	163.1/425.3/264.0	3.12	113	36/27/34	13
Apramycin (APR)	540.3	217.0/199.3	3.18	119	33/32	13
Dihydrostreptomycin (DHSTR)	584.3	263.2/246.2/221.1	3.06	70	42/49/38	15
Gentamicin (GEN)	478.0	322.0/157.0	3.21	55	21/23	13
Hygromycin B (HYG)	528.3	352.3/177.4	3.02	62	33/41	13
Kanamycin (KAN)	485.0	163.0/324.0/205.0	3.13	60	32/21/34	13
Neomycin (NEO)	615.1	161.0/163.0/293.0	3.25	130	37/38/33	15
Paromomycin (PAR)	616.1	163.2/293.2/324.0	3.19	110	40/30/21	13
Sisomicin (SIS)	448.3	254.3/271.3	3.19	96	33/26	13
Spectinomycin (SPC)	351.0	333.2/207.0	2.93	132	24/28	15
Streptomycin (STR)	582.0	263.0/246.0/221.0	3.06	153	44/50/40	15
Tobramycin (TOB)	468.3	163.0/145.0	3.20	67	29/25	13
Ribostamycin (RIB) *	455.0	163.0	3.12	71	30	13

* internal standard.

**Table 3:** Comparison of different SPE cartridges.

Matrix		SPE Cartridges		
	Strata X	Strata X-CW	Strata X-AW	Oasis HLB
Muscle	+	++	+	+++
Kidney	++	+	+	+++
Liver	+	++	++	+++
Fat	++	+	++	+++
Sausages	++	+	++	+++
Shrimps	+	+	+++	++
Fish	+	+	+++	++
Honey	+++	+	++	+++
Milk	++	+	++	+++
Eggs	+	++	+++	++
Whey powder	++	+	++	+++
Sour cream	+	+	++	+++
Curd	+	+	++	+++

+: not satisfactory (recovery −20–50%); ++: quite satisfactory (recovery −50–70%), +++: satisfactory (recovery −70–120%).

**Table 4:** Validation results.

Muscle							
Analyte	LOQ (µg/kg)	Validation Level (µg/kg)	Decision Limit CCα (µg/kg)	Repeatability *, (CV, %)	Within-Laboratory Reproducibility *, (CV, %)	Recovery * (%)	Matrix Effect * (CV, %)
AMI	25.0	50	59.4	6.24	4.72	102	3
APR	10.0	1000 *	1114	10.9	6.78	113	4
DHSTR	50.0	500 *	586	6.39	7.31	102	3
GEN	10.0	50 *	61.9	7.63	9.24	105	5
HYG	25.0	50	64.2	8.72	8.01	99.0	4
KAN	10.0	100 *	110	9.25	9.14	103	5
NEO	10.0	500 *	509	9.88	7.73	104	7
PAR	10.0	500 *	517	9.20	7.91	89.6	4
SIS	10.0	50	62.0	7.99	5.58	101	5
SPC	50.0	300 *	327	7.23	8.38	104	2
STR	25.0	500 *	543	13.9	13.1	101	3
TOB	10.0	50	56.7	7.27	5.44	103	3
**Kidney**							
AMI	10.0	50	58.1	9.91	6.21	108	5
APR	10.0	20,000 *	21,642	3.52	9.25	96.8	4
DHSTR	10.0	1000 *	1093	9.50	9.37	102	8
GEN	10.0	750 *	851	4.66	10.8	105	6
HYG	50.0	50	62.8	4.26	7.03	87.4	9
KAN	10.0	2500 *	2769	4.66	5.90	95.1	8
NEO	10.0	9000 *	10,632	12.5	10.8	109	4
PAR	10.0	1500 *	1740	9.59	7.19	113	5
SIS	10.0	50	53.5	8.86	5.65	107	5
SPC	25.0	5000 *	5967	4.31	5.79	92.3	3
STR	10.0	1000 *	1195	7.45	8.82	89.0	3
TOB	10.0	50	64.7	10.4	4.42	105	4
**Liver**							
AMI	50.0	50	63.0	13.0	11.8	104	6
APR	10.0	10,000 *	12,097	11.0	11.4	107	4
DHSTR	10.0	500 *	621	9.59	11.8	110	3
GEN	10.0	200 *	246	10.4	7.99	112	7
HYG	25.0	50	57.9	6.37	5.78	101	4
KAN	10.0	600 *	702	7.07	4.49	108	5
NEO	100	5500 *	5971	11.5	9.77	115	5
PAR	50.0	1500 *	1814	10.4	9.44	112	4
SIS	10.0	50	68.1	13.6	11.9	102	7
SPC	25.0	2000 *	2462	14.0	12.2	109	4
STR	10.0	500 *	594	12.0	11.4	103	6
TOB	10.0	50	69.9	10.3	8.88	105	5
**Fat**							
AMI	10.0	50	67.1	5.51	12.2	85.6	3
APR	10.0	1000 *	1194	4.28	5.78	107	4
DHSTR	10.0	500 *	537	5.75	9.02	87.2	8
GEN	10.0	50 *	61.3	9.28	7.75	109	6
HYG	10.0	50	69.5	8.05	8.26	106	4
KAN	10.0	100 *	117	6.97	4.49	97.3	6
NEO	10.0	500 *	604	7.08	8.25	101	8
PAR	10.0	50	51.8	8.95	7.12	88.5	6
SIS	10.0	50	63.7	5.58	6.44	89.4	6
SPC	25.0	500 *	563	9.43	5.78	100	7
STR	25.0	500 *	615	8.74	9.12	102	4
TOB	10.0	50	51.7	6.68	10.6	84.7	7
**Sausages**							
AMI	25.0	50	68.4	13.2	7.94	87.9	6
APR	25.0	50	64.7	10.3	9.68	82.8	4
DHSTR	25.0	50	53.8	9.84	10.7	94.5	7
GEN	10.0	50	56.6	7.98	9.58	102	6
HYG	50.0	50	67.9	8.85	10.3	106	5
KAN	10.0	50	57.3	11.3	9.36	97.2	5
NEO	25.0	50	51.8	4.71	6.13	96.5	4
PAR	25.0	50	70.3	7.09	9.15	99.4	5
SIS	10.0	50	54.9	10.8	11.4	107	5
SPC	25.0	50	55.8	11.7	10.9	115	6
STR	50.0	50	69.2	10.0	10.1	89.6	5
TOB	25.0	50	71.6	9.64	7.86	112	5
**Shrimps**							
AMI	10.0	50	68.7	7.68	9.12	103	8
APR	25.0	50	51.9	11.5	10.9	89.4	6
DHSTR	10.0	50	57.9	9.25	5.56	87.2	7
GEN	10.0	50	64.3	4.58	4.30	100	5
HYG	50.0	50	73.8	6.79	9.55	114	4
KAN	10.0	50	71.6	5.52	10.3	110	9
NEO	25.0	50	55.9	9.93	12.8	109	5
PAR	10.0	50	57.6	11.7	7.42	98.8	6
SIS	10.0	50	62.5	10.8	11.8	106	6
SPC	25.0	50	65.9	6.65	9.48	97.3	4
STR	50.0	50	58.9	7.48	10.1	89.5	4
TOB	10.0	50	74.9	5.43	12.6	118	9
**Fish**							
AMI	10.0	50	68.9	9.32	8.15	104	4
APR	10.0	50	54.3	7.45	9.37	87.0	4
DHSTR	10.0	50	55.4.	8.82	7.18	93.6	8
GEN	10.0	50 *	59.0	3.96	9.22	108	6
HYG	10.0	50	68.1	6.74	8.57	103	8
KAN	10.0	50	67.3	9.48	10.4	97.4	7
NEO	10.0	50	59.1	6.34	11.8	105	10
PAR	10.0	50	60.8	4.59	6.25	117	8
SIS	10.0	50	67.1	7.61	7.12	83.5	4
SPC	25.0	50	56.0	10.3	8.87	109	5
STR	25.0	50	71.3	9.78	7.56	89.4	10
TOB	10.0	50	54.7	7.42	10.7	101	5
**Honey**							
AMI	10.0	50	67.2	11.7	9.84	106	7
APR	10.0	50	70.6	10.7	11.5	110	3
DHSTR	10.0	50	68.4	5.29	4.71	110	8
GEN	10.0	50	68.8	9.15	7.41	95.4	9
HYG	50.0	50	57.3	8.76	5.59	91.2	4
KAN	10.0	50	65.5	11.9	8.36	109	6
NEO	10.0	50	69.0	5.40	2.71	86.3	6
PAR	10.0	50	57.1	4.62	4.15	114	6
SIS	10.0	50	59.7	3.11	10.5	107	6
SPC	10.0	50	67.3	8.27	7.71	89.7	4
STR	10.0	50	52.9	12.0	9.05	98.1	4
TOB	10.0	50	57.1	8.26	5.95	106	5
**Milk**							
AMI	10.0	50	71.3	10.4	8.54	111	5
APR	25.0	50	59.0	4.82	5.70	103	5
DHSTR	10.0	200 *	261	7.62	6.88	105	5
GEN	10.0	100 *	127	7.48	8.43	98.4	6
HYG	25.0	50	60.8	8.58	6.92	105	7
KAN	10.0	150 *	172	5.52	6.13	103	6
NEO	10.0	1500 *	1690	10.0	8.66	100	8
PAR	10.0	50	67.6	9.28	9.39	110	6
SIS	10.0	50	57.9	5.57	8.74	105	5
SPC	50.0	200 *	262	9.08	9.96	109	9
STR	10.0	200 *	257	10.1	8.74	104	6
TOB	10.0	50	64.8	4.83	4.54	99.2	12
**Eggs**							
AMI	10.0	50	71.9	7.03	7.97	109	5
APR	10.0	50	64.2	4.55	5.02	102	8
DHSTR	10.0	50	57.8	8.01	8.70	108	8
GEN	10.0	50	60.8	8.94	9.96	108	15
HYG	50.0	50	67.1	8.92	9.41	97.3	7
KAN	10.0	50	55.3	6.74	10.1	102	7
NEO	10.0	500 *	587	9.47	10.8	112	8
PAR	10.0	200 *	229.0	11.7	11.2	108	13
SIS	10.0	50	57.1	10.0	9.60	101	8
SPC	50.0	100	131	4.79	6.44	88.6	8
STR	10.0	50	58.3	7.37	5.13	110	5
TOB	10.0	50	68.1	5.22	5.81	103	13
**Whey Powder**							
AMI	25.0	50	65.8	8.57	11.2	114	5
APR	50.0	50	58.1	6.99	10.8	94.3	7
DHSTR	10.0	50	63.0	8.80	6.25	96.5	11
GEN	10.0	50	67.1	10.2	9.13	87.4	7
HYG	100	100	118	8.43	5.70	85.9	5
KAN	25.0	50	54.6	10.8	8.24	107	8
NEO	100	250	271	12.0	10.6	104	7
PAR	25.0	50	56.3	9.16	7.89	116	8
SIS	10.0	50	58.1	8.96	8.54	97.8	6
SPC	50.0	100	128	10.0	11.8	100	6
STR	50.0	100	116	11.8	13.8	93.7	5
TOB	10.0	50	55.8	8.67	9.60	99.1	8
**Sour cream**							
AMI	50.0	50	55.3	9.13	10.9	87.6	4
APR	50.0	50	57.2	7.64	8.46	91.4	3
DHSTR	25.0	50	60.8	10.8	7.68	109	7
GEN	25.0	50	64.1	9.64	12.5	99.8	6
HYG	50.0	100	115	8.25	9.36	106	6
KAN	25.0	50	54.9	7.55	10.5	117	7
NEO	250	250	279	6.84	11.3	108	10
PAR	50.0	100	128	14.2	8.56	94.7	6
SIS	50.0	50	57.3	9.46	9.97	96.8	7
SPC	50.0	100	121	8.67	7.56	83.7	4
STR	50.0	100	119	9.12	10.6	100	8
TOB	50.0	50	57.6	9.45	11.7	110	7
**Curd**							
AMI	100	100	109	4.56	9.10	83.9	8
APR	25.0	50	54.3	8.12	7.54	102	7
DHSTR	10.0	50	57.8	11.6	7.49	96.4	5
GEN	50.0	50	60.9	5.79	10.3	84.0	8
HYG	100	100	117	6.45	7.96	88.7	6
KAN	50.0	50	65.7	10.5	9.81	103	13
NEO	100	250	262	9.86	8.30	118	8
PAR	100	100	117	8.64	8.76	105	8
SIS	10.0	50	65.9	10.8	11.3	96.1	6
SPC	50.0	100	124	13.5	7.99	94.7	6
STR	25.0	100	131	8.25	10.1	83.0	6
TOB	10.0	50	54.3	7.51	9.36	87.5	9

* MRL.

## Data Availability

Not applicable.

## Supplementary Materials

The following supporting information can be downloaded at: https://www.mdpi.com/article/10.3390/molecules28124595/s1, Figure S1: The effect of different pH values: a—pH = 5.5; b—pH = 6.5; c—pH = 8.0 in (A) muscle, (B) kidney, (C) fat, (D) sausages, (E) shrimps, (F) fish, (G) milk, (H) eggs, (I) whey powder, (J) sour cream, (K) curd.
