# Supplementary material for: Analysis of Aminoglycoside Antibiotics: A Challenge in Food Control
Source: Molecules. 2023 Jun 7;28(12):4595. doi: 10.3390/molecules28124595 (PMC10301657; doi:10.3390/molecules28124595)
Supplement: Supplementary file 1 [file molecules-28-04595-s001.zip › molecules-2411283-supplementary.pdf]

Analysis of Aminoglycoside Antibiotics: A Challenge in Food Control

Ewelina Nowacka-Kozak *, Anna Gajda and Małgorzata Gbylik-Sikorska

Department of Pharmacology and Toxicology, National Veterinary Research Institute, Partyzantów 57, 24-100 Puławy, Poland

* Correspondence: ewelina.nowacka@piwet.pulawy.pl; Tel.: +48-81-889-3415

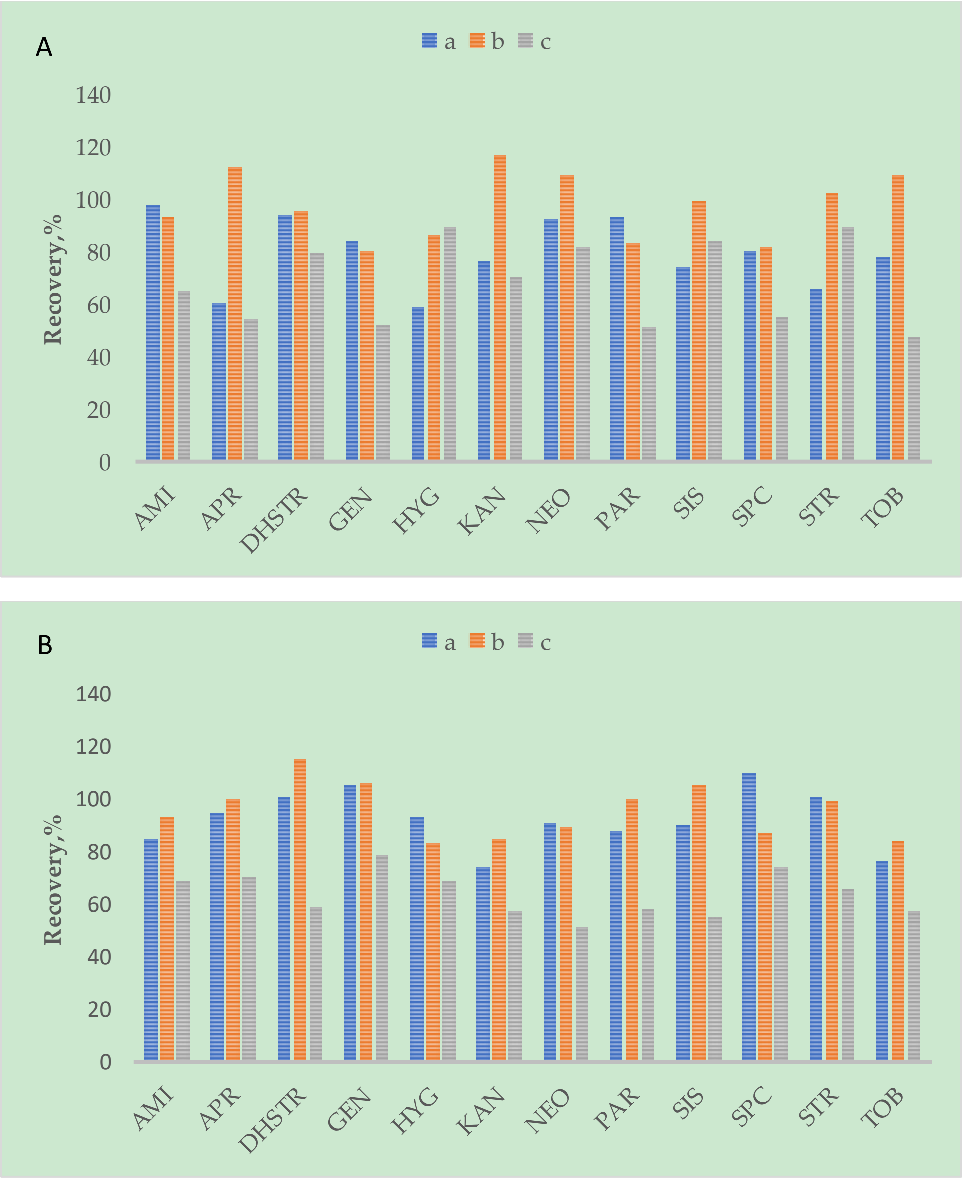

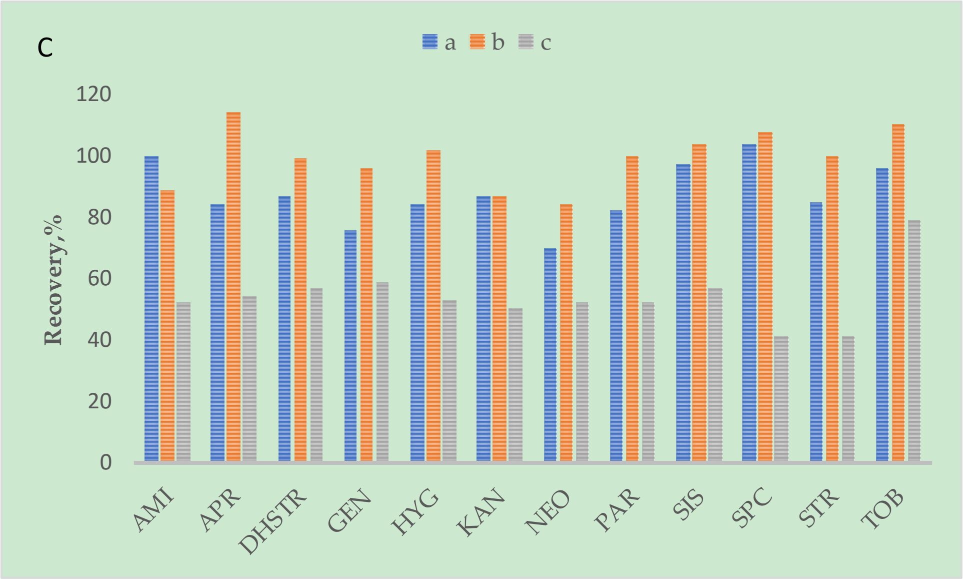
C
a b c
Recovery,%
0
20
40
60
80
100
120
AMI
APR
DHSTR
GEN
HYG
KAN
NEO
PAR
SIS
SPC
STR
TOB

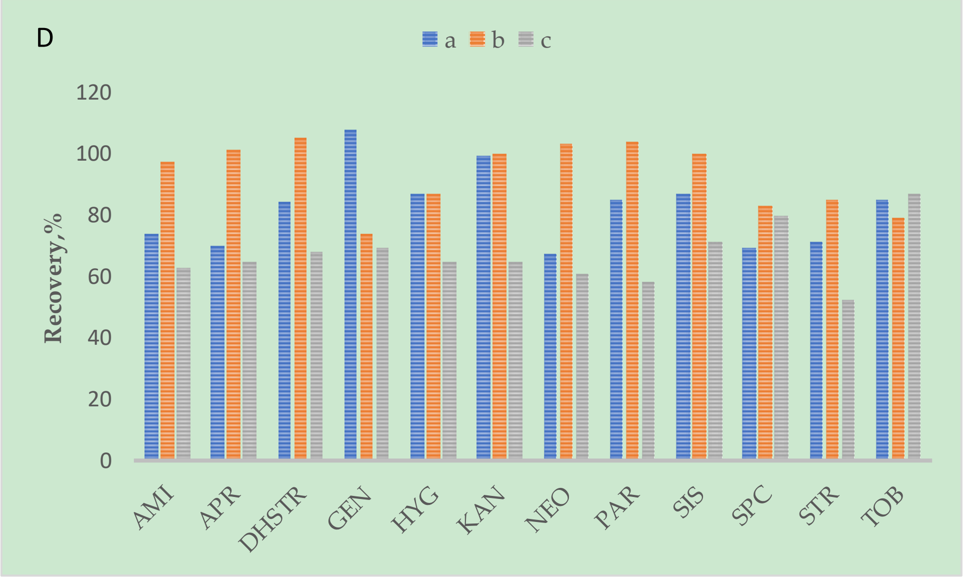
D
a b c
Recovery,%
0
20
40
60
80
100
120
AMI
APR
DHSTR
GEN
HYG
KAN
NEO
PAR
SIS
SPC
STR
TOB

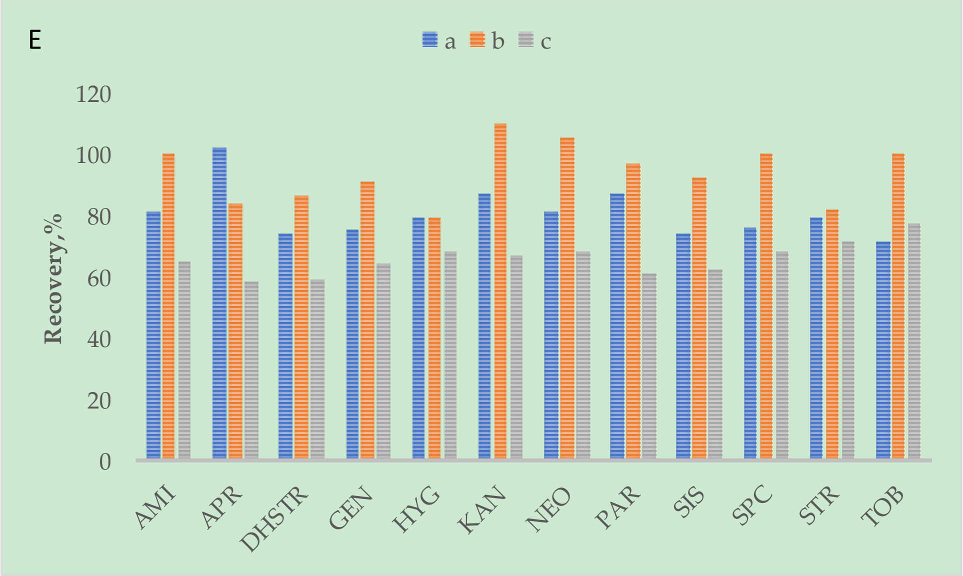
E
a b c
Recovery,%
0
20
40
60
80
100
120
AMI
APR
DHSTR
GEN
HYG
KAN
NEO
PAR
SIS
SPC
STR
TOB

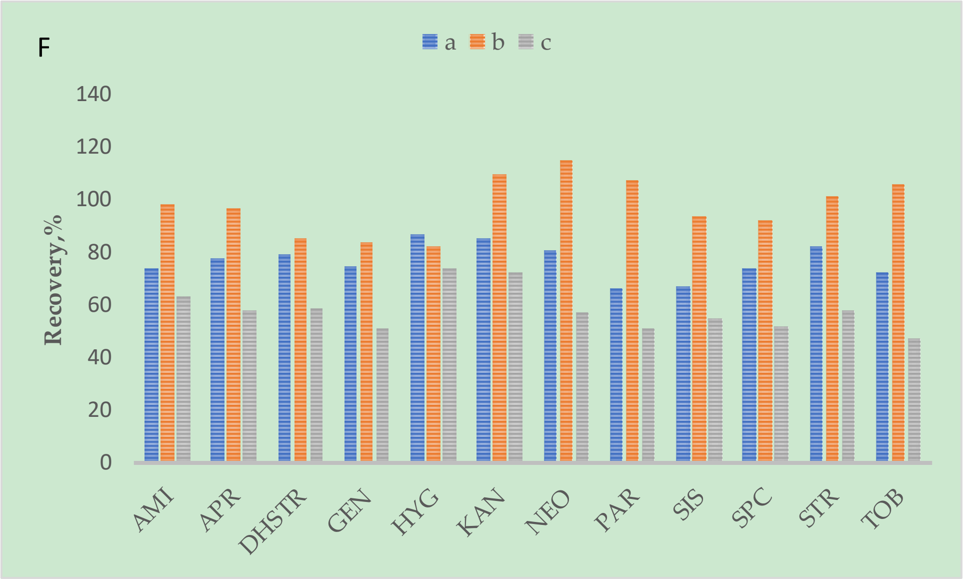
F
a b c
Recovery,%
140
120
100
80
60
40
20
0
AMI
APR
DHSTR
GEN
HYG
KAN
NEO
PAR
SIS
SPC
STR
TOB

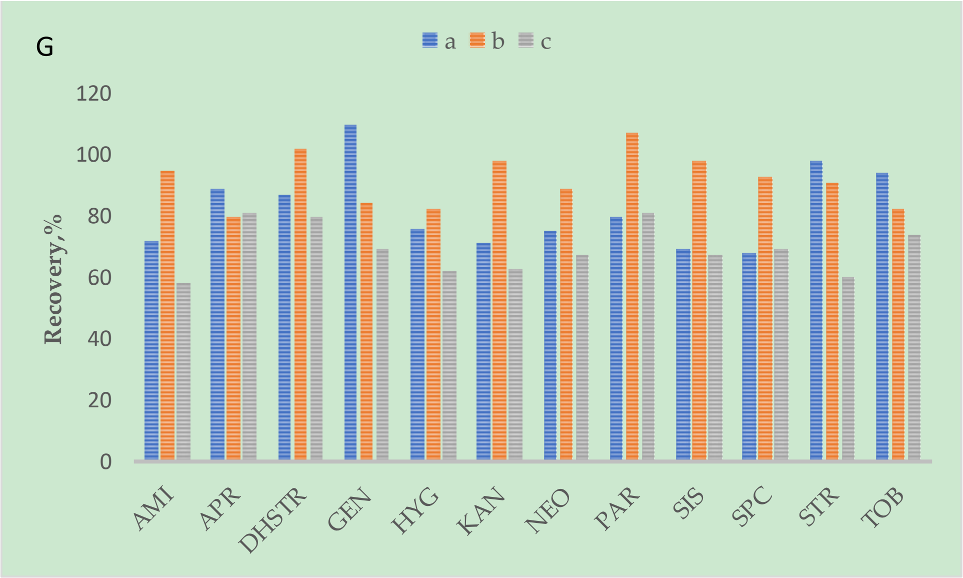
G
a b c
Recovery,%
120
100
80
60
40
20
0
AMI
APR
DHSTR
GEN
HYG
KAN
NEO
PAR
SIS
SPC
STR
TOB

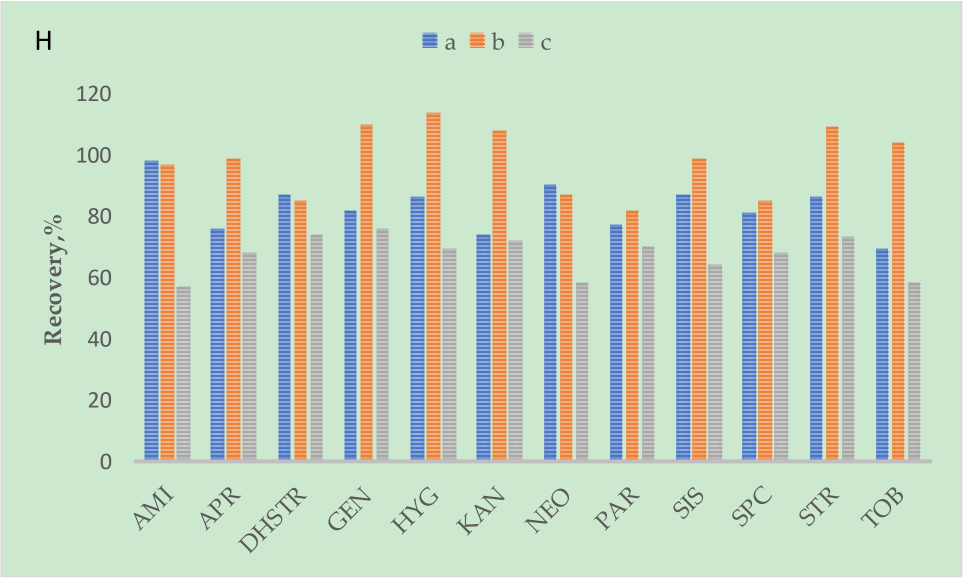
H
a b c
Recovery,%
120
100
80
60
40
20
0
AMI
APR
DHSTR
GEN
HYG
KAN
NEO
PAR
SIS
SPC
STR
TOB

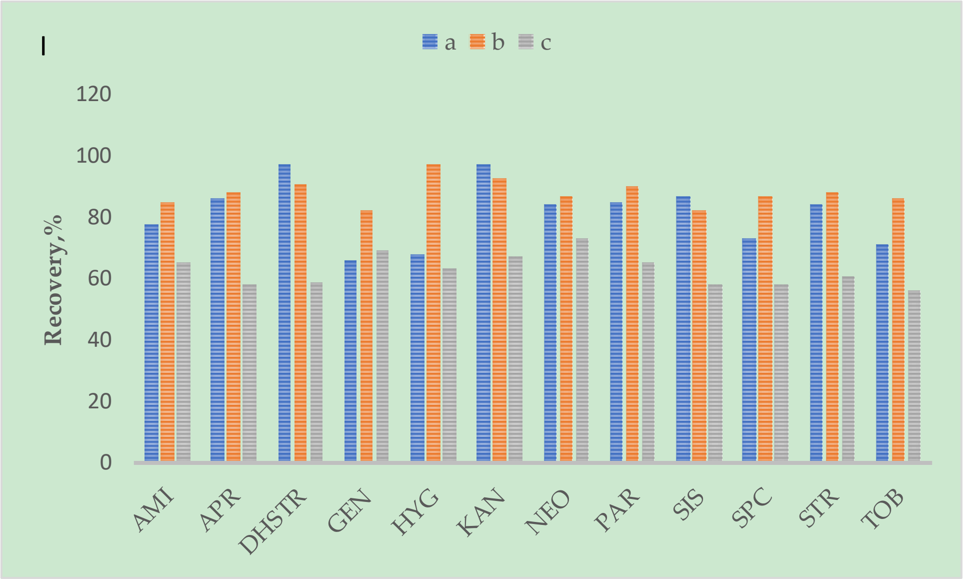
I
a b c
Recovery,%
120
100
80
60
40
20
0
AMI
APR
DHSTR
GEN
HYG
KAN
NEO
PAR
SIS
SPC
STR
TOB

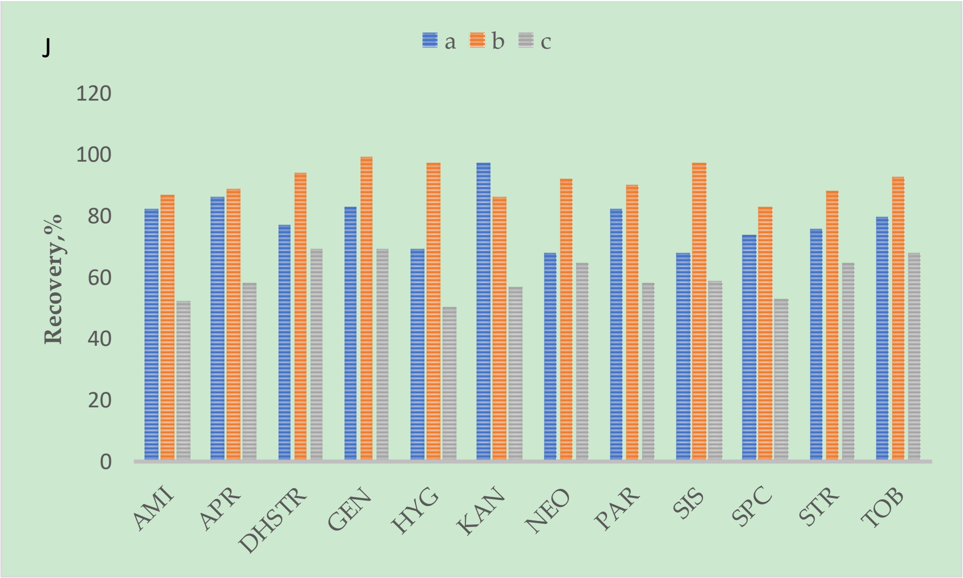
J
a b c
Recovery,%
120
100
80
60
40
20
0
AMI
APR
DHSTR
GEN
HYG
KAN
NEO
PAR
SIS
SPC
STR
TOB

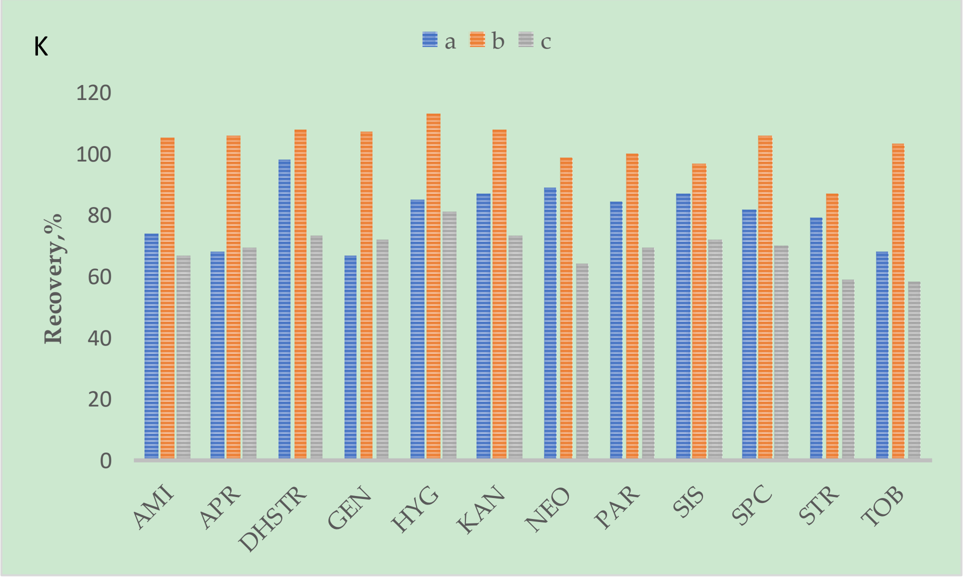
K
a b c
Recovery,%
120
100
80
60
40
20
0
AMI
APR
DHSTR
GEN
HYG
KAN
NEO
PAR
SIS
SPC
STR
TOB

Figure S1. The effect of different pH values: a - pH=5.5; b - pH=6.5; c - pH=8.0 in (**A**) muscle, (**B**) kidney, (**C**) fat, (**D**) sausages, (**E**) shrimps, (**F**) fish, (**G**) milk, (**H**) eggs, (**I**) whey powder, (**J**) sour cream, (**K**) curd.
